# Simultaneous High-Grade Upper-Tract Urothelial Carcinoma and Xanthogranulomatous Pyelonephritis

**DOI:** 10.7759/cureus.111100

**Published:** 2026-06-18

**Authors:** Sohail Nakhuda, Adedeji Fatuga, Grace Elliott, Laxmi Radhakrishnan, Ata Jaffer

**Affiliations:** 1 Urology, Stepping Hill Hospital, Manchester, GBR; 2 Urology, Manchester Medical School, Manchester, GBR; 3 Pathology, Stepping Hill Hospital, Manchester, GBR

**Keywords:** nmibc (non-muscle-invasive bladder cancer), unilateral nephrectomy, upper-tract urothelial carcinoma, urothelial malignancy, xanthogranulomatous inflammation, xgp

## Abstract

A 67-year-old female with a previous history of superficial urothelial carcinoma of the bladder, discharged from surveillance follow-up, presented to her general practitioner with nausea and unintentional weight loss. The patient had radiological findings of a poor nephrogram with significant perirenal inflammatory stranding and hydronephrosis secondary to conglomerate pelvic calcification. This was suggestive of xanthogranulomatous pyelonephritis (XGP) and possible malignant changes. The patient ultimately underwent a radical nephrectomy; subsequently, the specimen revealed high-grade T4 urothelial cell carcinoma with background XGP, with vascular invasion and positive margins, but no evidence of spread to regional lymph nodes. She was referred onwards to oncology for further treatment given her margin status and is undergoing an intense and prolonged course of surveillance follow-up. This is likely one of the first reported cases of advanced disease with such diagnostic difficulty and surgical complexity.

## Introduction

Upper-tract urothelial carcinoma (UTUC) and xanthogranulomatous pyelonephritis (XPG) are individually infrequent presentations, with concurrent occurrences being exceptionally rare. This case details a 67-year-old female in whom high-grade renal urothelial carcinoma (UC) and XGP were simultaneously suspected, with postoperative histology confirming both. There have been limited reported instances of coexisting UTUC and XGP cases [1-3], though none presenting with such advanced disease and surgical complexity.

UC is a malignancy originating from the transitional cells lining the urinary tract system. While predominantly arising in the bladder, 5-10% of urothelial tumors are found in the upper urinary tract [1], with up to 2 cases per 100,000 yearly [4]. Smoking stands out as the primary risk factor, with additional associations noted with exposure to occupational carcinogens, heavy coffee consumption, and cyclophosphamide.

XGP is an exceptionally uncommon variant of granulomatous response seen in chronic pyelonephritis in the setting of chronic obstruction from renal calculi. It constitutes fewer than 1% of cases [5], with an annual incidence of 1.4 per 100,000 [6]. XGP can be focal, segmental, or diffuse, with the latter being the most prevalent variant, where it can progress through nephric, perinephric, and paranephric stages in its natural history. Patients suffering from diffuse XGP often suffer extensive unilateral renal injury due to the destructive nature of the granulomatous process damaging the parenchyma. *Escherichia coli* and *Proteus mirabilis* are the isolated agents in approximately 90% of XGP-positive urine cultures [7].

Initial diagnosis typically involves CT imaging, with confirmation through gross pathological and microscopic examination of nephrectomy specimens, with radical nephrectomy being the recommended treatment for advanced-stage disease. Histologically, the findings in XGP include lipid-filled macrophages (foamy histiocytes) in the background of inflammatory findings.

Most cases of XGP are unilateral; therefore, a surgical solution is optimal. However, bilateral disease has been reported and is usually fatal [8], though bilateral nephrectomy with long-term dialysis is an option. Fortunately, most cases have a favorable prognosis with prompt treatment, as will be discussed in this case.

## Case presentation

A 67-year-old female with a prior history of superficial non-muscle-invasive (G2 pTa) UC of the bladder was treated and discharged after surveillance with no recurrence. She re-presented to her general practitioner in January 2024 with nausea and unintentional weight loss, but no visible hematuria. A CT of the abdomen showed abnormalities in the right kidney (Figure 1), and a CT urogram with contrast revealed marked irregularities, with a poor quality nephrogram, significant perirenal inflammatory stranding, calcification, and hydronephrosis (Figure 2). No metastatic disease was detected on CT or nuclear medicine bone scan.

**Figure 1:**
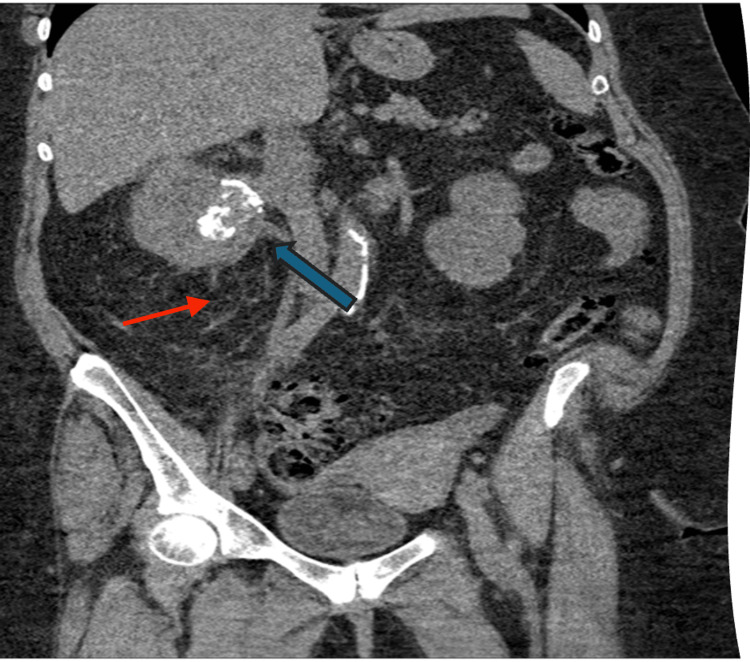
Coronal CT scan showing the right kidney with significant calcification and narrowing of the upper ureter leading to hydronephrosis. The blue arrow shows the narrow upper ureter with hydronephrosis proximally. The red arrow shows perirenal inflammatory fat stranding.

**Figure 2:**
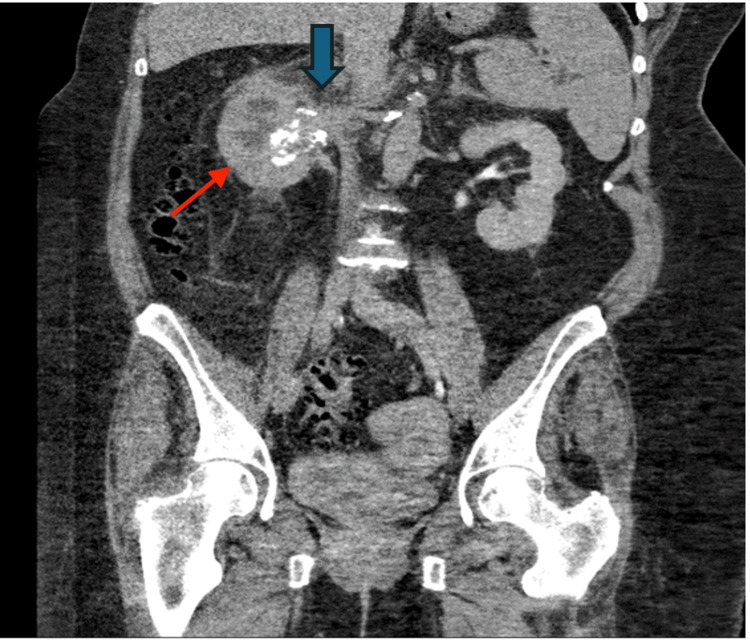
Delayed urogram image showing normal excretion from the contralateral left kidney and no excretion from the dilated right kidney. The blue arrow shows the adherent inferior vena cava and inflammatory changes of the right kidney. The red arrow shows cortical thinning of the right kidney.

The appearance of the left kidney was not concerning, with no visible lesions and good opacification on administration of contrast. Renal function was good with the estimated glomerular filtration rate (eGFR) being 67 mL/min/1.73 m^2^. Inflammatory markers were slightly raised with a C-reactive protein of 63.7 mg/L and a white cell count of 9.2 × 10^9^/L. Urine cytology was suggestive of acute inflammation with no high-grade malignant cells seen. Urine culture showed pyuria but no growth of organisms. These results raised suspicion of inflammatory change with potential underlying malignancy, with a right radical nephroureterectomy proposed during the urology multidisciplinary team discussion, due to the high likelihood of a UTUC. Following a discussion with the patient in the clinic, they opted for a radical nephroureterectomy, as biopsy of the kidney/lesion was unfortunately not an option due to significant inflammation seen on imaging.

The patient underwent the procedure in February 2024. It began as a right robotic radical nephrectomy with flexible cystoscopy; however, it was converted to an open radical nephrectomy following the discovery of significant inflammatory changes around the hilum, causing complete adherence to the inferior vena cava (IVC). The adrenal gland was spared, and the distal ureter was clipped at the iliac bifurcation, obviating full nephroureterectomy. Two lumbar veins were taken, sutures were applied to the lateral aspect of the IVC inferior to the hilum, and the offending organ was successfully excised (Figure 3). Complete nephroureterectomy was not possible due to the difficulty encountered with the specimen and the risk of significant blood loss and adjacent organ injury.

**Figure 3:**
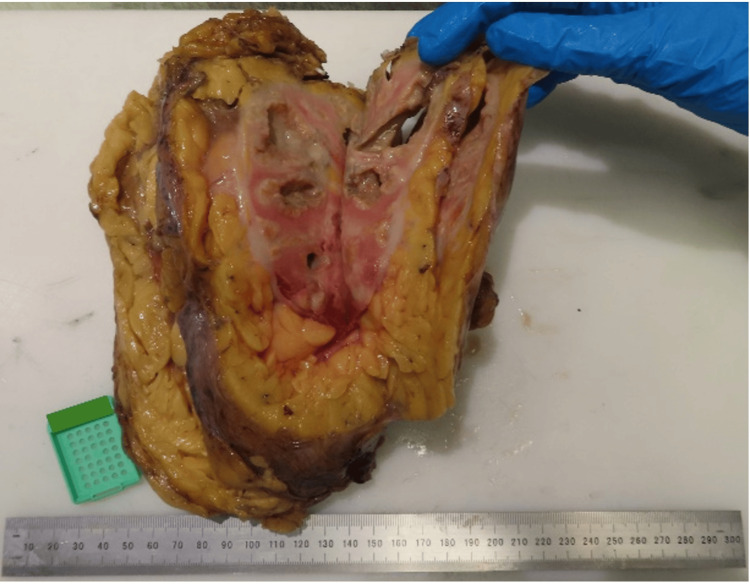
Morphological picture of the nephrectomy specimen. Note the loss of corticomedullary differentiation and yellowish irregular surface.

The histological findings showed a renal pelvis high-grade (WHO 2004 classification) UC, which infiltrated extensively into the renal parenchyma and the perinephric fat (Figure 4), with background XGP (Figure 5). Vascular invasion was present (Figure 6) along with perineural invasion (Figure 7), and the margins were positive at the hilum. There was no nodal disease or metastatic spread, and final staging was determined to be T4 N0 M0 R1.

**Figure 4:**
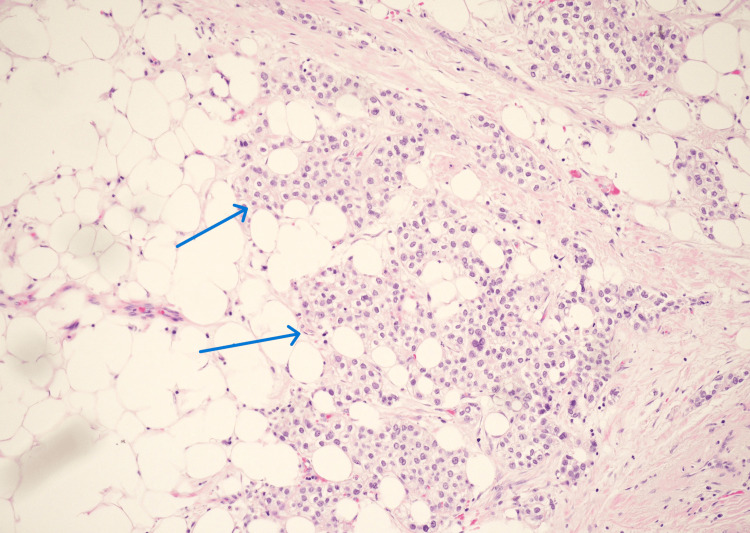
Perinephric fat invasion. The arrows indicate the tumor infiltrating perinephric fat.

**Figure 5:**
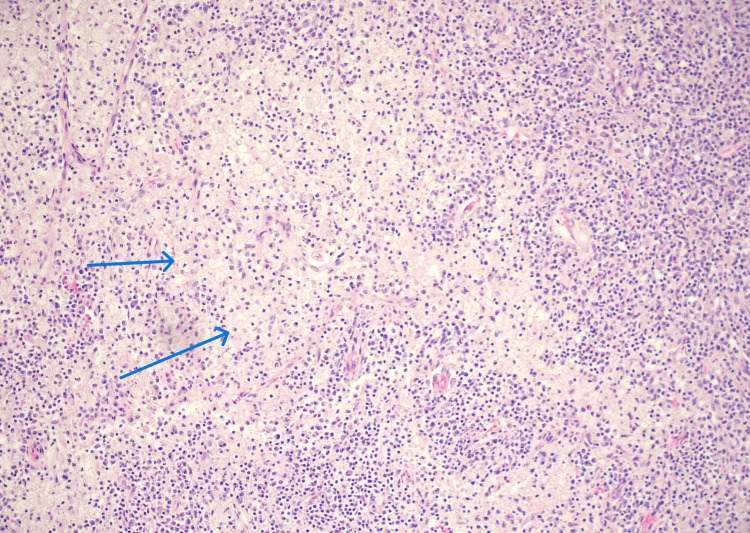
Xanthogranulomatous pyelonephritis. The arrows annotate sheets of foamy histiocytes.

**Figure 6:**
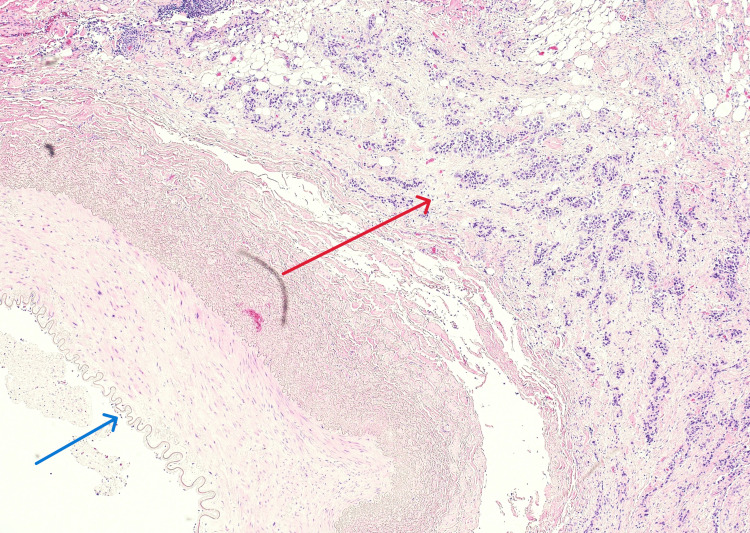
Vascular invasion. The blue arrow indicates internal elastic lamina of vessel, and the red arrow annotates the tumor invading the vessel wall.

**Figure 7:**
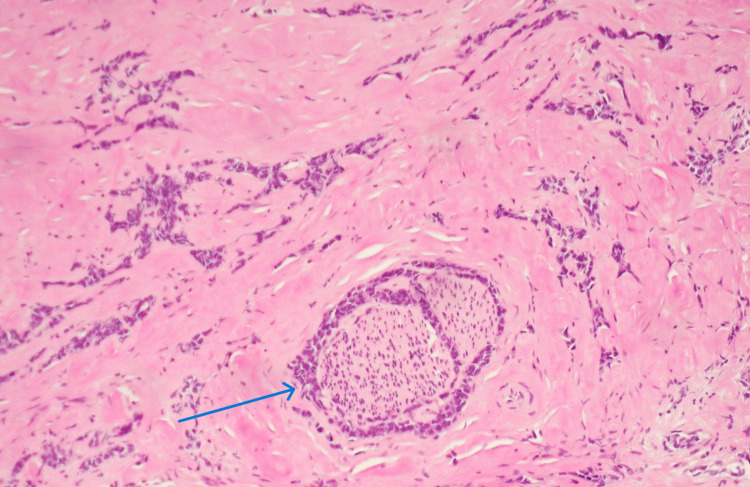
Perineural invasion. The blue arrow indicates the tumor infiltrating a nerve twig.

Following the operation, the patient was admitted for a short stay in the intensive care unit and commenced the postoperative protocol of low-molecular-weight heparin, while continuing her own regular medications. She was later moved to the ward after spending two nights in intensive care, and was then discharged to her home on postoperative day seven, with no requirement for additional community care. There was no readmission within 30 days of discharge, and her post-nephroureterectomy eGFR was 49 mL/min/1.73 m^2^ from her previous 67 mL/min/1.73 m^2^, indicating good contralateral kidney function.

She was then referred to the oncologist after the multidisciplinary team meeting for adjuvant chemotherapy due to high stage and grade of disease. She was informed of her positive margin status and counselled about the possible risk of progression of her disease. She was also planned for surveillance cystoscopies and CT imaging as per the European Association of Urology recommendations for high-risk UTUC with previous non-muscle-invasive bladder cancer. The patient has remained cancer-free to date with no recurrence at flexible cystoscopy or on CT imaging.

## Discussion

This patient’s case is distinctive in that she presented with simultaneous UTUC and XGP, both rare phenomena and exceedingly so in combination. There have been cases reported that show an association with renal cell carcinoma; however, we could find only one other case where UTUC was found on histological analysis [3]. Conversely, in our case, UTUC was suspected from the initial presentation due to the patient’s background of bladder cancer, and appropriate management of nephroureterectomy was planned but unfortunately failed, as discussed.

UC is an inherently multifocal disease with a recognized propensity for recurrence after initial treatment [4], making this a potential explanation considering the patient’s previous experience with non-muscle-invasive bladder cancer, and this was a strong consideration in this index presentation.

Comorbidities must remain a consideration. Our patient suffered from long-standing type 2 diabetes mellitus, a condition which has been outlined as a risk factor for both XGP and UC [8-10]. There have also been reports of development of these conditions following long-term inflammation and obstruction within the urinary system following recurrent urinary tract infections. These factors could explain the patient’s predisposition to both illnesses, though not necessarily indicate a causal relationship between the two.

While CT urography remains the gold standard for evaluating UTUC, radiological features of XGP, such as renal enlargement, parenchymal destruction, perinephric stranding and poor contrast enhancement, could resemble advanced urothelial or renal cell carcinoma [11]. There have also been reports of UTUC clinically mimicking inflammatory renal pathology [12], in which nephrectomy was performed for infective obstruction, with high-grade UTUC only revealed on histological investigation.

Urologists must also be aware of the surgical complexity that can be encountered in this cohort of patients. XGP is well recognized for causing dense inflammatory adhesions that may involve the renal hilum, surrounding vessels, or adjacent structures. In our patient, this necessitated conversion from a robotic to an open approach; hence, patients must be counselled on this appropriately. Furthermore, full nephroureterectomy was unable to be performed; hence, oncological control may be compromised, requiring adjuvant treatment and close surveillance protocols. Long-term follow-up has not been recorded as of yet, and this is a limitation we acknowledge.

XGP is often treated with conservative percutaneous drainage; however, these cases reinforce the importance of open-minded management, particularly in patients with comorbidities and declining renal function. Given the difficulty in diagnosis based on imaging, as the true diagnosis may not be found until histological analysis, underlying malignancy should be considered in these patients for appropriate planning and counselling.

## Conclusions

This case highlights a rare and life-threatening presentation of concurrent UTUC and XGP in an older female patient. Greater awareness of the possible associations between inflammatory pathology and malignancy would help physicians diagnose and manage patients appropriately. Early suspicion and diagnosis may help facilitate timely intervention, appropriate oncological management, and improved patient outcomes.
